# Structure-Related Optical Characteristics of Thin Metallic Films in the Visible and Ultraviolet

**DOI:** 10.6028/jres.080A.064

**Published:** 1976-08-01

**Authors:** H. E. Bennett, J. L. Stanford

**Affiliations:** Michelson Laboratories, Naval Weapons Center, China Lake, California 93555

**Keywords:** Absorption, dielectric layers, irregularities, metallic thin films, microirregularities, overcoating, plasmons, scatter

## Abstract

Surface irregularities and crystalline order strongly influence both the scattered light and absorption of metallic films. These effects extend through all spectral regions but are particularly important in the visible and ultraviolet. Scattered light arises from several scattering mechanisms. Macroscopic irregularities such as dust, scratches and particulates are typically much less important than are microirregularities only a few tens of angstroms in height but covering the entire surface. For metals such as silver and aluminum, which have plasma edges in the ultraviolet, the excitation of surface plasmons resulting from these microirregularities causes additional incoherently reemitted or “scattered” light. Surface plasmon excitation also causes increased absorption in some wavelength regions. These effects are enhanced by dielectric overcoating layers, which both increase the absorption and scattering and shift the wavelength at which the peak occurs. Surface plasmon excitation is particularly important in the ultraviolet region, where the dielectric overcoating applied to prevent formation of an oxide film on aluminized mirrors, for example, can significantly change the mirror reflectance. Plasmon excitation is made possible by a momentum conserving process associated with material inhomogeneities and hence can presumably be caused by crystalline disorder in the metal surface as well as surface irregularities. If the disorder is present on a sufficiently fine scale, it also affects the band structure of the metal and hence its optical absorption. Examples of the effect of film structure on the optical properties of evaporated and sputtered metal films will be given.

## I. Introduction

The optical characteristics of opaque metal films which are of most interest are the fraction of the light which is either absorbed or scattered by the films. The most commonly assumed mechanism for light scattering consists of reflection from numerous tiny scratches, dust particles and other surface blemishes which act as tiny mirrors oriented at various angles to the surface normal. This picture has marginal merit in the infrared, where scattering from particulates and surface blemishes usually is dominant, but is not the important mechanism for light scattering from optical surfaces in the visible and ultraviolet regions of the spectrum. To the extent that it is a geometrical optics phenomenon, scattering from surface blemishes such as scratches will be independent of wavelength. Particulate scattering, however, is largely a diffraction phenomenon. Closely related is resonant scattering and absorption associated with the polarization characteristics of individual irregularities in the metal surface. To the approximation that these irregularities are independent, i.e., that the scattering from one irregularity is unaffected by the presence of its neighbors, we may compute the resultant scattering and absorption approximately from the Mie scattering theory. Particulate scattering and scattering from large “macroirregularities*’’* can usually be regarded in this way. However the dominant source of scattered light for good optical surfaces in the visible and ultraviolet regions is closely spaced microirregularities only a few tens of angstroms in height. These microirregularities scatter and absorb collectively and cannot be regarded as independent. They can be treated statistically, and give rise to several interesting phenomena.

When monochromatic electromagnetic waves are incident on a smooth metal surface they induce virtual surface currents within the skin depth of the metal. The currents are virtual since the electrons in the metal have too much inertia to couple to the wave, but they have the same wave vector along the surface as the incident wave. Thus the radiation from these currents must also have the same wave vector along the surface as the incident wave, and the radiation leaves the surface in the specular direction. When surface irregularities are present, the current distribution is modulated according to the distribution of surface heights as well as their correlation along the surface. Additional tangential components are thus introduced into the wave vector of the current distribution producing nonspecular or scattered light. If we confine ourselves to total integrated scatter (TIS) and ignore its angular dependence or polarization at nonnormal incidence, the scattering caused by perturbation of the virtual current distribution can be described by classical diffraction theory in the Kirchhoff integral approximation. This effect is the most important source of scattered light in the visible and ultraviolet wavelength regions.

Actual surface currents can exist in the skin depth of the metal but their allowed phase velocity is lower than that of the virtual currents induced on a smooth surface by an incident light wave and thus no coupling occurs. However when microirregularities are present, coupling can in some cases occur and these surface charge waves can absorb energy from the incident light. These surface charge waves are quantized into units termed surface plasmons with a fixed energy-wave vector relationship. Once excited they may decay thermally into single electron excitations or they may radiate because of their acceleration along the curved surface irregularities. Surface plasmons are strongly affected by the presence of a dielectric layer on the metal surface. Both absorption and scattering are often greatly enhanced, a result which has significant implications since protective dielectric overcoating layers must be used to prevent oxidation of aluminum reflectors in the ultraviolet or sulfide formation on silver reflectors used at longer wavelengths. To minimize surface plasmon related absorption and scattering in such cases the mirror must be extremely smooth.

Other structure-related mechanisms also affect the optical properties of metals. Both mechanical abrasion and the inherent disorder in polycrystalline films of vacuum evaporated or sputtered metals result in increased absorption. The crystalline imperfections in such metal samples modify the electron energy band structure, permit excitation of surface plasmons by the incident electromagnetic fields, and affect the motion of conduction electrons. In the latter case the effect is greatest in the infrared, where the optical properties of metals are determined by the mean free path and number of conduction electrons.

In the visible and ultraviolet the response of single electrons in metals to incident light is influenced significantly by interband electron transitions. Because the energy band structure results directly from the regularity of the lattice structure, lattice distortion produced by mechanical abrasion or polycrystalline film growth affects the optical response of metals. The optical properties of metals thus prepared are notoriously nonreproducible in the visible and ultraviolet. In addition to single electron effects, the inhomogeneities resulting from the disorder permit absorption via excitation of collective electron oscillations, namely, surface plasmon excitation. The combination of these two effects can significantly reduce the reflectance of metals in the visible and ultraviolet through increased absorption.

Surface irregularities can also affect the mean free path of the conduction electrons near the metal surface. The conduction electrons have a de Broglie wavelength associated with their energy and may be thought of as a wave motion incident on the metal surface from inside. If the surface is perfectly smooth they reflect specularly and there is no change in their mean free path between collisions, i.e., the electron-surface interaction does not represent a collision in the specular case. If the conduction electrons are diffusely reflected, however, as they will be if surface microirregularities are present which are not adequately screened by the surface electron cloud, reflection from the surface does represent a collision and the electronic mean free path will be decreased with consequent increase in absorption. This “anomalous skin effect” [1]^1^ is important in the infrared region of the spectrum [2].

Surface microirregularities as well as macroirregularities may modify the optical properties of a metal surface through resonant scattering and absorption of light associated with resonance effects in the polarization characteristics of individual irregularities. These effects are equivalent in many ways to scattering from small dispersed particles and may be calculated in the first approximation using Rayleigh or Mie scattering theory.

In this paper we will first discuss briefly the theoretical predictions for optical absorption and scattering in metal surfaces due to structure-related mechanisms. Those which appear to be most important in the visible and ultraviolet regions are scattering and absorption from isolated microirregularities, and diffraction effects from correlated irregularities including the optical excitation of surface plasmons. We will then discuss instrumentation for measuring scattering, absorption, and surface structure, and finally will show some experimental results for silver and aluminum surfaces.

## II. Theory

### A. Light Scattering and Absorption by Isolated Irregularities

If we assume that we can treat each scattering center independently, the scattering may be computed using the Mie theory. Consider first non-absorbing irregularities. Assuming spherical scattering centers and defining *x* as *x* = 2*πa*/*λ* where *a* is the particle radius, *λ* the wavelength and *n* the index of refraction, the ratio *Q_s_* of scattering to geometrical cross section of the scatterer for *nx* <0.8 is [3]

$$Q_{s}=\frac{8}{3}x^{4}{\left(\frac{n^{2}-1}{n^{2}+2}\right)}^{2}\left[1+\frac{6}{5}\left(\frac{n^{2}-1}{n^{2}+2}\right)x^{2}+\cdots \right].$$ (1)

**Figure 1:**
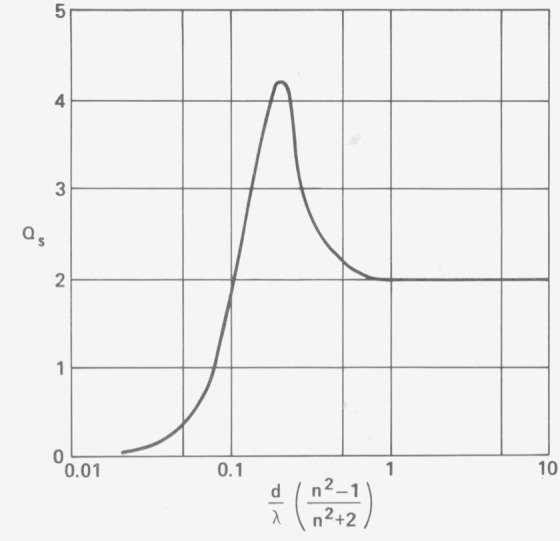
Theoretical prediction of scattering cross section *Q*_s_ as a function of the ratio of particle diameter d to wavelength λ times a function of index of refraction. The graph is calculated for spherical, nonabsorbing particles. The effective area of each scattering center is πd^2^Q_s_/4.

The Rayleigh 1/*λ*^4^ law for *x* << 1 follows from eq (1). For *x* >> 1, *Q_s_* = 2. In the region *x* ~ 1 sometimes complicated resonance occurs. E. D. Bailey [4] has suggested the universal scattering curve shown in fig. 1 as a first approximation to this resonance. If the index of refraction of the scattering center is in the 1.3–1.4 range, the resonance then occurs when the diameter of the scattering center is approximately equal to the wavelength. Dust particles are probably largely nonabsorbing.

If the index of refraction of the scattering center is complex, as would be the case if scattering were occuring from an asperity on the metal surface rather than from a dust particle, both absorption and scattering would occur. The ratio *Q_e_* of extinction cross section to geometrical cross section, which includes both scattering and absorption, would then be given by [3]

$$Q_{e}=-Im\left[4x\left\{\frac{{\tilde{n}}^{2}-1}{{\tilde{n}}^{2}+2}+\frac{4}{15}x^{3}{\left(\frac{{\tilde{n}}^{2}-1}{{\tilde{n}}^{2}+2}\right)}^{2}\frac{{\tilde{n}}^{4}+27{\tilde{n}}^{2}+38}{2{\tilde{n}}^{2}+3}\right\}\right]+x^{4}\frac{8}{3}{\left|\frac{{\tilde{n}}^{2}-1}{{\tilde{n}}^{2}+2}\right|}^{2}$$ (2)

where the complex index *ñ = n−ik* and *k* is the extinction coefficient. The term in brackets gives the absorption and the second term the scattering as in eq (1) except that now *ñ* is complex. A resonance would still occur in this case, but as *k* increases the short wavelength side of figure 1 will fall off less rapidly than for the nonabsorbing case.

### B. Scalar Scattering Theory

**Figure 2:**
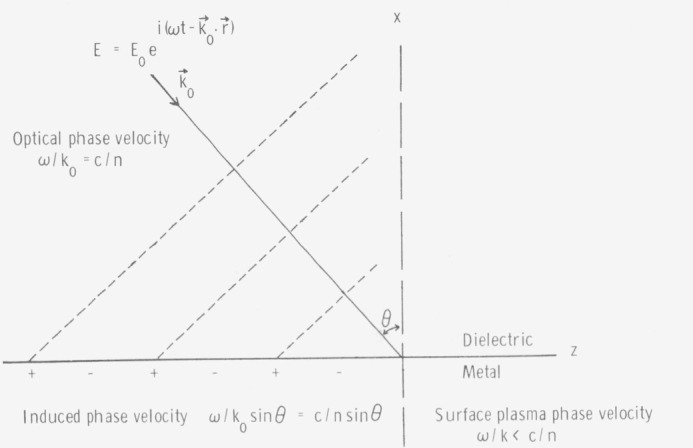
Surface charge wave induced by the electric field of an incoming electromagnetic wave. The dashed lines indicate maximum field strength. The induced charge wave is virtual since its phase velocity is ≥ the velocity of light, and a real surface plasma wave must have a phase velocity ≤ c/n.

In addition to particulates and other isolated irregularities on an optical surface, there are also closely spaced irregularities, often having very small deviations from the mean surface level (MSL), which scatter and absorb collectively. The effect of these interdependent irregularities may be understood by considering the surface current induced by an incident electromagnetic wave. Figure 2 illustrates the surface charge wave which would be induced if the electrons in the metal could move rapidly enough. These charge waves are virtual states under most conditions since the charge density waves which can actually exist within the skin depth of a metal have phase velocities *ω*/*k*_0_ which are less than the velocity of light *c*/*n* in the dielectric in contact with the metal surface, whereas, as seen from figure 2, the phase velocity of induced surface charge waves is greater than *c*/*n.* The component of the wave vector of this induced virtual surface charge wave is *k*_0_ sin *θ* where *k*_0_ is the wave vector of the incident light and *θ* the angle of incidence. The radiation from this induced current must also have the same wave vector component along the surface in order to satisfy the boundary conditions associated with the fields. Therefore, the radiation from the surface leaves in the specular direction. However if irregularities were present on the surface, the current distribution would be modified according to the heights of these irregularities as well as their correlation along the surface. Additional tangential components are thus introduced into the wave vector of the current distribution producing nonspecular or “scattered” light.

**Figure 3:**
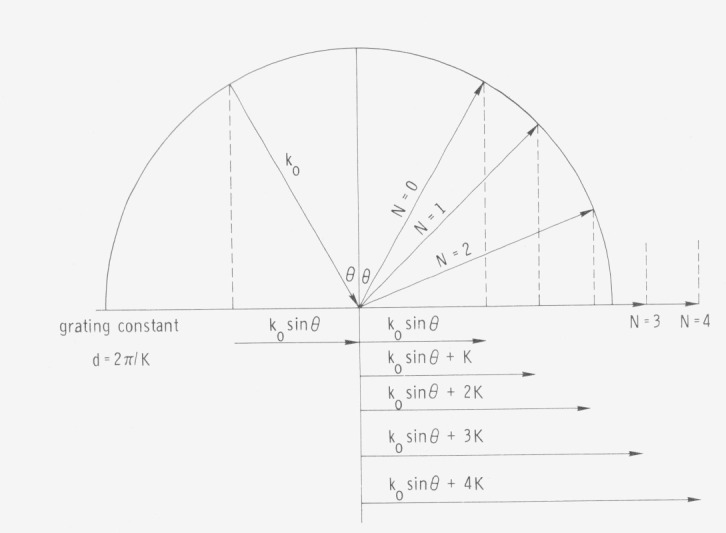
Momentum transfer from a grating surface to a light beam. *κ*_0_ is the vector of the incident light, N the order of interference, *d* the line spacing on the grating, and K the wave vector for the grating surface.

An example of the effect of correlated surface irregularities on light reflected from a surface is furnished by a reflection grating with groove spacing *d* shown in figure 3. The wave vector ***K*** associated with the grating grooves is *K* = 2*π*/*d.* If light with wave vector ***k***_0_ is incident on the grating, in the *N* = 0 order, the light is reflected specularly and has a component of its wave vector along the surface of *k*_0_ sin*θ* as in the case for the smooth surface. In other orders the magnitude of the wave vector is unchanged (i.e., the wavelength of light reflected in various directions is the same) but the component of the wave vector along the surface of the grating is increased by multiples of ***K***, the wave vector associated with the grating surface. Photons in the light incident on the grating have energies of *ħω* and momenta of *ħ****k***_0_. The photons reflected from the grating have the same energy but the component of their momenta along the surface has been altered by integral multiples of the momentum *ħ****K*** associated with the grating surface. The effect of a rough surface can thus be interpreted as supplying an additional tangential momentum component to the reflected photon without changing its resultant energy or total momentum *ħ****K***_0_ *= ħω*/*c.* The momentum which the surface can supply is determined by the statistical character of its surface irregularities.

The angular dependence and polarization of scattered light requires an involved theoretical analysis [5, 6]. However, if we confine ourselves to total integrated scatter from irregularities which are large laterally relative to the wavelength of light, the scattering caused by perturbation of the virtual surface current distribution can be described quite simply by classical diffraction theory in the Kirchhoff integral approximation[7–9]. A statistical representation of the surface irregularities is sufficient to make the calculation. Of primary importance is the height distribution function of surface irregularities about the MSL. Interferometric analyses using fringes of equal chromatic order (FECO interferometry) indicate that the height distribution function of real optical surfaces is very nearly Gaussian[10]. For a surface with a Gaussian height distribution the ratio of the specularly reflected light *R* to the total reflectance *R*_0_ is[8]

$$\frac{R}{R_{0}}=e^{-{\left(4\pi \delta /\lambda \right)}^{2}}$$ (3)

where *δ* is the rms value of the height of surface microirregularities about the MSL. Equation (3) is exact [9] for all values of *δ*/*λ* and expresses the fraction of the reflected light which is reflected coherently from different points on the surface. However as *δ*/*λ*→1 an increasing amount of incoherently reflected or scattered light falls very near to the specular direction and cannot be separated from it experimentally. Therefore eq (3) is most useful in describing the experimentally observed scattered light for *δ*/*λ* << 1. In this case the exponent may be expanded, giving the scattered light Δ*R* as

$$\Delta R=R_{0}{\left(4\pi \delta /\lambda \right)}^{2}.$$ (4)

Two precautions must be observed in using eq (4): (1) the *rough* surface total reflectance must be used for *R*_0_. It may or may not be equal to the smooth surface reflectance, and (2) scattering from surface plasmon excitation and decay must be excluded, i.e., scattering measurements to determine *δ* must be taken in wavelength regions where surface plasmon excitation is not important.

**Figure 4:**
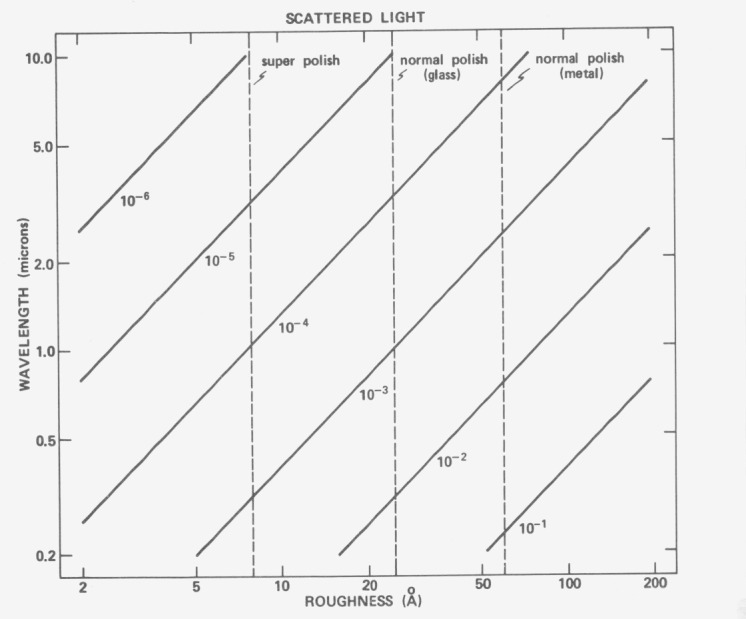
Theoretical predictions for scattering as a function of roughness and wavelength.

Figure 4 shows the fractional scattering Δ*R/R*_0_ predicted by eqs (3) and (4) as a function of wavelength and surface roughness. The solid diagonal lines give the theoretically predicted scattering levels. The dashed vertical lines show typical roughness values for superpolished optical surfaces, high quality glass optics and high quality metal optics polished by conventional techniques. In the photographic infrared typical metal coated glass mirrors will scatter about 0.1 percent of the reflected light. This value quadruples in the visible region and in the near ultraviolet approaches 1 percent. At shorter wavelengths it increases still further, although in the vacuum ultraviolet the situation is unclear. In the intermediate and far infrared scattered light caused by microirregularities becomes quite low and other types of scattering become important.

### C. Optical Excitation of Surface Plasmons

The “scalar scattering theory” described above predicts a monotonic increase in scattering with decreasing wavelength. Several years ago we and others [11–13] found that slightly rough metal surfaces can exhibit resonant absorption in the spectral range near the metal’s surface plasmon frequency. Accompanying this resonant absorption is increased scattering which also occurs predominantly in the surface plasmon region. These phenomena may be interpreted in terms of optical excitation of surface plasmons, [14], which then decay either thermally (absorption) or radiatively (scattering). Surface plasmons may be excited optically in either thin metal films, where the surface oscillations at the two surfaces can couple, or in thick films (i.e., significantly thicker than the optical penetration depth of the light, which for the best conductors is a few tens of nm). We will consider only the thick film case here.

The allowed surface charge waves which may exist on a conducting surface may be obtained directly from Maxwell’s equations, which may be written in Gaussian units as [15]

∇⋅D=0 (5)

∇⋅H=0 (6)

$$\nabla \times E=-\frac{1}{c}\frac{\partial H}{\partial t}$$ (7)

$$\nabla \times H=i\frac{\omega }{c}(\epsilon_{1}-i\epsilon_{2})E$$ (8)

where *ϵ*_1_ and *ϵ*_2_ are the real and imaginary parts of the complex dielectric constant *ϵ* and the other symbols have their usual significance. Choosing a co-ordinate system at the metal-dielectric interface in which *x* is directed into the metal along the surface normal and *y* and *z* are in the plane of the interface, Maxwell’s equations are satisfied by the transverse magnetic (TM) wave solution

$$E=E(x)e^{i(w^{t}-k_{0}z)}$$ (9)

where *E_y_* = *H_x_* = 0 at *x* = 0. From eqs (5), (8) and the wave equation given by ∇ **×** ∇ **×** *E*, we obtain

$$E_{z}=-\frac{i}{k_{0}}\frac{dE_{x}}{dx}$$ (10)

$$H_{y}(x)=\frac{\omega \epsilon }{ck_{0}}E_{x}$$ (11)

$$\frac{d^{2}E_{x}}{dx^{2}}-\left(k_{0}^{2}-\frac{\omega^{2}\epsilon }{c^{2}}\right)E_{x}=0.$$ (12)

Solving eq (12) gives

$$\begin{array}{l} E_{x}=E_{m}e^{-{\left(k_{0}^{2}-\omega^{2}\epsilon_{m}/c^{2}\right)}^{1/2_{x}}}\;x>0\\ =E_{d}e^{+{\left(\kappa_{0}^{2}-\omega^{2}\epsilon d^{/c^{2}}\right)}^{1/2_{x}}}\;x<0 \end{array}$$ (13)

where subscripts *m* and *d* refer to the metal and dielectric respectively. *E_z_* and *H_y_* can be calculated from E*_x_* using eqs (10) and (11). The TM surface charge waves are evanescent with phase velocity *ω*/*k*_0_. Boundary conditions are that *ϵE_x_* and *dE_x_*/*dx* are continuous across the metal-dielectric interface, giving

$$k_{0}=\frac{\omega }{c}{\left(\frac{\epsilon_{m}\epsilon_{d}}{\epsilon_{m}+\epsilon_{d}}\right).}^{1/2}$$ (14)

Equation (14) is a general relation giving the wave vector of the surface charge wave in terms of the complex dielectric constants of the metal and dielectric. Note that a resonance can occur if *ϵ*_1_= − *ϵ_d_* where *ϵ_m_* = *ϵ*_1_−*iϵ*_2_ and that it will be strongest when *ϵ*_2_≅0 i.e., damping is minimized. For real metals *ϵ_m_* is a complicated function. However, for a free electron metal with no damping

$$\epsilon_{m}=\epsilon_{1}=1-{\omega_{p}}^{2}/\omega^{2}$$ (15)

where *ω_p_* is the plasma frequency. Substituting in eq (14) we obtain the dispersion curve for surface charge waves at a free electron metal-air interface:

$$\omega =\omega_{p}{\left\{0.5+{\left(k_{0}/k_{p}\right)}^{2}+{\left[0.25+{\left(k_{0}/k_{p}\right)}^{4}\right]}^{1/2}\right\}}^{1/2}$$ (16)

where *k_p_ = ω_p_*/*c.* Letting *k*→∞ we see that there will be a maximum frequency *ω_sp_* which the plasmon can have where

$$\omega_{sp}=\omega_{p}/{\left(1+\epsilon_{d}\right)}^{1/2}.$$ (17)

For a metal-air interface, then, the maximum frequency which surface plasmons in a free electron metal can have is $\omega_{p}/\sqrt{2}$.

**Figure 5:**
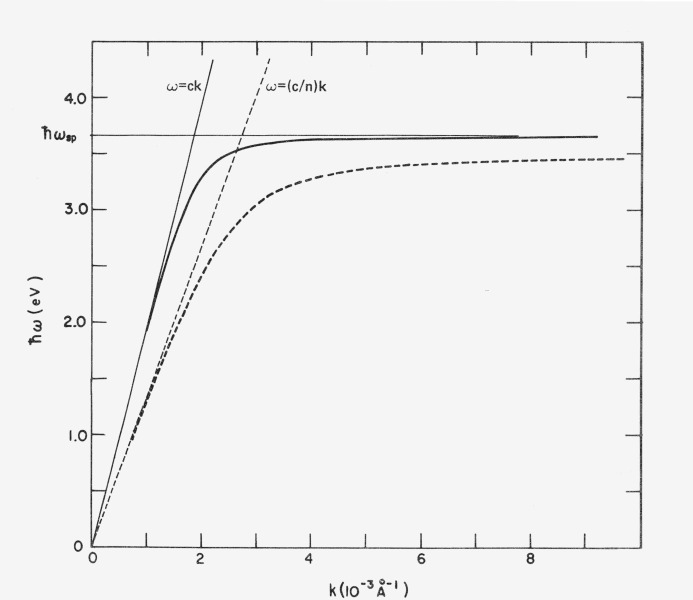
Dispersion curves for light waves and surface plasma waves at a metal-dielectric interface where the dielectric has a refractive index of unity (solid lines) and an index of n > 1 (dashed lines). Surface wave modes exist for all values of ω below a limiting frequency ω_sp_ but the density of states is highest near ω_sp_. As the index increases ω_sp_ decreases and the k values for the surface waves remain larger than those associated with the light waves.

Equation (16) is plotted in figure 5 as the solid line. Note that the *k*_0_ value for the surface plasma waves is always larger than that of the incident light waves having the same energy and becomes very much larger near the limiting frequency. The density of allowed states is highest in this energy region. The momentum which must be furnished by the surface for coupling to occur is, for normally incident light, all of that to the left of the surface plasma wave dispersion curve, since the wave vector of normally incident light has no component parallel to the surface and the wave vector of the surface wave is entirely in the surface. For grazing incidence light, only the difference between the “light line” *ω* = *ck k*_0_ and the dispersion curve need be supplied and in the “retardation region” where *ω*<<*ω_sp_* they approach coincidence. In the grating illustrated in figure 3 an order of interference could occur for which light would be diffracted at a grazing angle. If the frequency of the light were in the retardation region, coupling could then occur and energy would be absorbed by the grating from the beam to excite surface plasma waves. This situation does occur and is the origin of the Woods anomalies in diffraction gratings. [11, 16].

A dispersion curve analogous to eq (16) can also be calculated from eqs (14) and (15) when the dielectric is not air. Such a curve is shown in figure 5 as the dashed line with its associated “light line.” The effect of increasing *ϵ_d_* is to lower *ω_sp_* and move the frequency associated with a given *k*_0_ to lower values. Thus when a dielectric is deposited on a metal surface without affecting the resultant *k*_0_ values, we would expect resonances in absorption or scattering to move to longer wavelengths.

**Figure 6:**
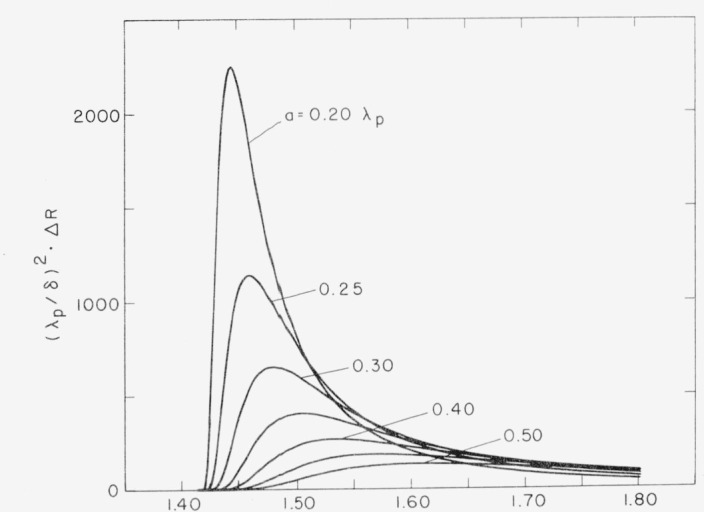
Absorption in a free electron metal caused by surface plasmon excitation. λ_p_ is the limiting wavelength for surface plasmon excitation, a the autocovariance length and δ the rms surface roughness, both assuming a Gaussian model, and ΔR the increase in absorption for wavelength λ. Calculations are based on the Crowell-Ritchie model.

Calculating the coupling probability between incident photons and surface waves resulting from surface roughness is a difficult quantum mechanical problem. It has been attacked by Elson and Ritchie [17] by means of a perturbation calculation in which damping is neglected. They obtain the probability that a photon of energy *ħω* incident normally on a rough surface will be converted into a surface plasmon, which may be written as the decrease in reflectance of the specular beam Δ*R_sp_* where

$$\Delta R_{sp}=\delta^{2}{\left(\frac{\omega }{c}\right)}^{4}\frac{{\epsilon_{1}}^{2}}{{\left[-(1+\epsilon_{1})\right]}^{5/2}}g\left(\frac{\omega }{c}{\left[\frac{\epsilon_{1}}{1+\epsilon_{1}}\right]}^{1/2}\right)$$ (18)

where *g* is the surface structure factor, which is the two dimensional Fourier transform of the autocovariance function of the surface roughness. In general, as the autocovariance length *a* of the surface irregularities becomes longer the momentum spectrum of surface roughness becomes more concentrated toward the origin in *k*_0_ space and the probability for photon-surface plasmon conversion becomes lower. Thus the optical excitation of surface plasmons will be relatively weak for gently rolling surfaces and much stronger for more jagged surfaces. In addition it will increase with the square of the rms height of surface irregularities and will also increase as the wavelength of the incident light decreases. Theoretical absorption curves calculated from eq (18) for optical excitation of surface plasmons on a free electron metal surface having Gaussian height distribution and autocovariance functions are shown in figure 6 for various values of *a.* As *a* becomes larger relative to *λ_p_* the peak absorption decreases but the wavelength range over which an effect occurs becomes longer. Thus although the magnitude of effect is less for a gently rolling surface than for a jagged one, the wavelength range over which the effect may be important should increase.

## III. Experimental Procedure

In order to accurately measure scattering and absorption in the visible and ultraviolet regions and to relate them to surface structure, various nonstandard instruments are required. Four types of instruments which have been of particular help at Michelson Laboratory are the NWC Scatterometer and the Optical Evaluation Facility for measuring scattered light, the NWC Absolute Reflectometers for measuring reflectance, and the NWC FECO Scanning Interferometer for determining the structure of optically polished surfaces. A brief description of these instruments follows.

### A. NWC Scatterometer

**Figure 7:**
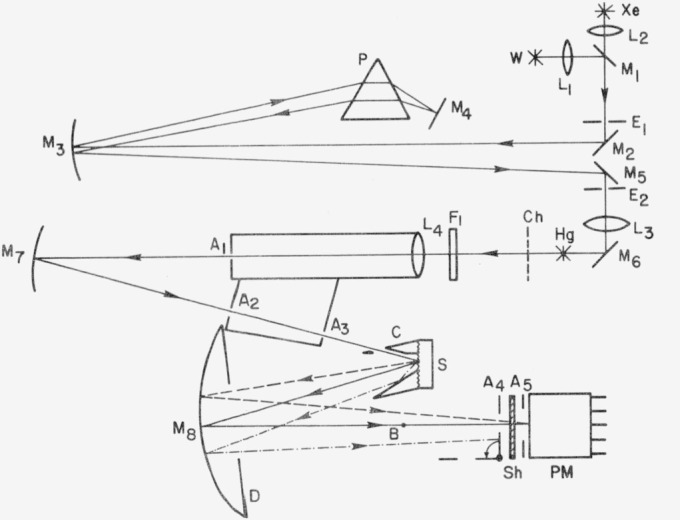
Schematic diagram of Scatterometer. The symbols are defined in the text.

Figure 7 gives a schematic diagram of the NWC Scatterometer [18]. Mirrors are indicated by M, lenses by L, slits by E, apertures by A, the adjustable diaphragm by D, the photomultiplier by PM, and its shutter by Sh, and the dispersing prism by P. In operation dispersed light from either a high pressure xenon arc Xe or a tungsten filament W is used. Alternatively, a low pressure mercury arc Hg may be used as a monochromatic source with the aid of filters F. The light is collimated and a pencil beam 1 mm in diameter passes through A_3_ to strike sample S, a plane circular sample 3.86 cm in diameter, at an angle of incidence of 15°. Specularly reflected light passes out along the axis of internally aluminized cone C of semivertex angle 18°45′ and is reflected by M_8_ to PM. It may be entirely blocked by B, which is situated at the focus of A_3_ and removes all light within a half angle of 37′ of the specular direction. Light scattered within 20° of the specular direction strikes M_8_ directly and is focussed at A_5_. At larger scattering angles light is reflected from C to M_8_, which then images the virtual object formed by reflection from C as a ring which can be blocked off by rotating aperture A_4_. By manipulating B, D, and A_4_ light scattered into all angles or into a range of angles between 37′ and 1½° to 20°, may be ratioed to total sample reflectance. Advantages of this instrument are that it uses mirror optics and thus is nearly independent of wavelength, it does not rely on having a Lambertian surface as does an integrating sphere, and it is sufficiently efficient to measure scattering levels down to below 10^−5^ when care is taken to ensure that the detector and electronics are linear over this dynamic range. A three polarizer unit [19] is helpful for such linearity investigations.

### B. Optical Evaluation Facility

**Figure 8:**
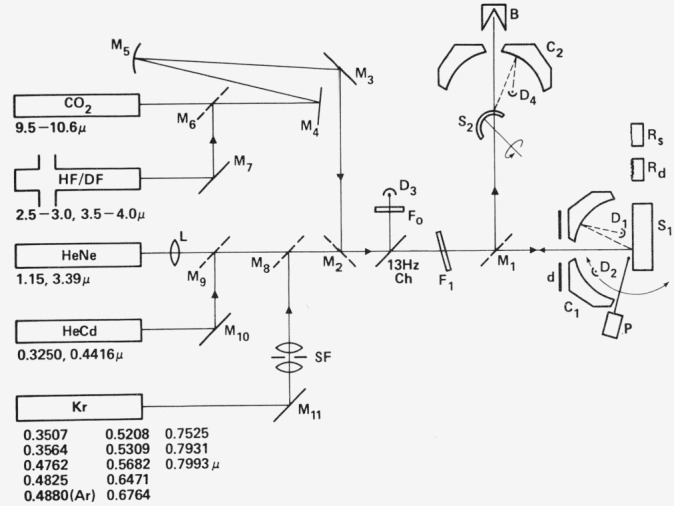
Schematic diagram of the Optical Evaluation Facility showing lasers and available laser lines, mirrors M, lens L, spatial filter SF, attenuation filters F, chopper Ch, diaphragm d, beam collector B, Coblentz spheres C, pyroelectric detectors D, samples S and R.

For measuring scattered light from flat or curved samples of various shapes up to 40 cm in diameter the Optical Evaluation Facility shown schematically in figure 8 is useful. This instrument, which is somewhat more straightforward in concept than the Scatterometer, utilizes Coblentz spheres, indicated by C, and pyroelectric detectors D to measure scattered light in either reflection (for opaque samples) or transmission (for semitransparent ones). Back-scattering from transparent samples can also be measured. Approximate reflectance measurements can be made by using for comparison a reflectance standard *R_s_* that has been calibrated in the absolute reflectometer. Over 20 laser lines ranging from the ultraviolet through the intermediate infrared are available, and by utilizing rotatable detector *D*_2_ bidirectional reflectance distribution function (*BRDF*) measurements may also be taken. In addition to the pyroelectric detector a silicon detector for added sensitivity in the visible region is mounted on *D*_2_. Sample movement and data reduction are automated and computer controlled so that a sample surface can be scanned automatically to determine scattering levels as a function of position on the surface.

### C. NWC Absolute Reflectometer

**Figure 9:**
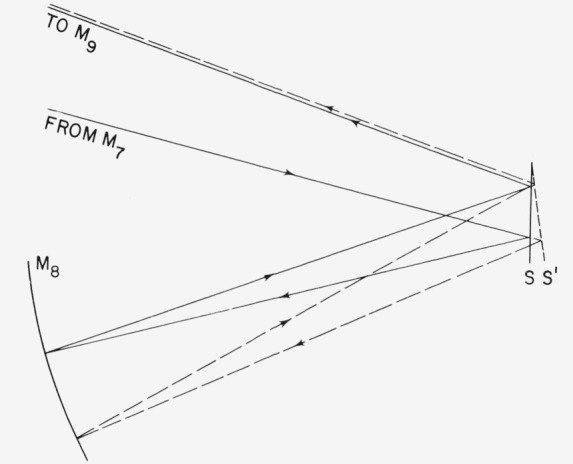
Principle of double reflection absolute reflectometer showing cancellation of beam deviation caused by sample tilt.

For making absolute spectral reflectance measurements the principle shown in figure 9 is employed. Light is reflected at nearly normal incidence from the sample S to a spherical mirror whose center of curvature is at the center of the sample surface. The mirror reimages the beam on the sample where it is again reflected and passes out to the detector. This scheme has several advantages. Most important is cancellation of beam deviation resulting from sample tilt. If the sample is tilted to S′, as shown in the figure, the beam is deviated through twice the tilt angle and strikes mirror M_8_ at a slightly different place. A spherical mirror used at its center of curvature images an object at the same place regardless of where the light beam from the object strikes the mirror surface, so M_8_ reimages at the same point for position S′ as it does for position S. Since the sample is plane, on the second reflection the beam tilt introduced on the first reflection is exactly cancelled. M_8_ has a long focal length to give a relatively large effective *f* number, so that both S and S′ are within the focal range of the mirror. Thus, in our reflectometers one can reach in, tilt the sample manually and observe no change in the beam at the detector position even when it is viewed with a high power microscope. In this way the most common cause of systematic error in reflectance measurements is eliminated.

**Figure 10:**
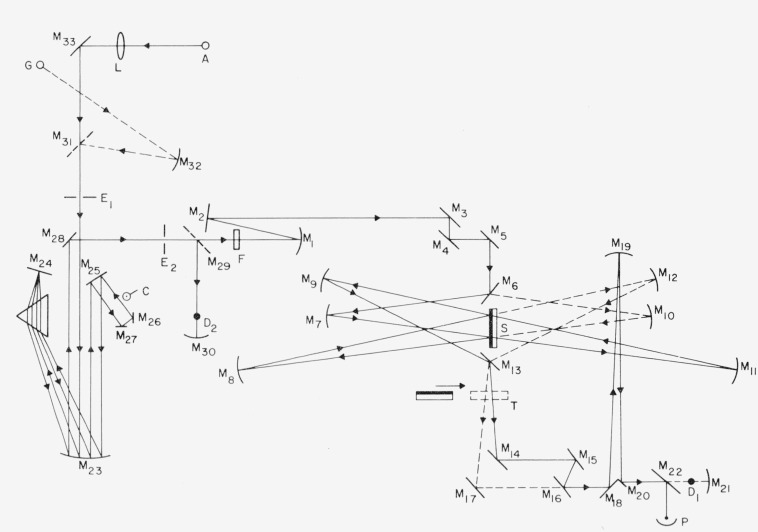
Schematic diagram of absolute reflectometer for use in visible and infrared wavelength regions employing principle illustrated in figure 9: sample S, transmission sample T, mirrors M, lens L, thermocouple detectors D, photomultipler P, slits E, chopper C, visible light source A, globar (infrared source) G, and filter F.

Figure 10 shows a schematic diagram of one of our three absolute reflectometers [20, 21]. The sample is mounted at S and can be removed from the beam, in which case light falls on M_11_, a mirror nearly identical to M_8_, and returns to follow the same optical path as for the sample-in position. The optical path length is the same for sample-out and sample-in, so no differential atmospheric absorption can occur and the same areas of the same mirrors are used for both cases with the exceptions of M_8_ and M_11_. By optically interchanging them by rotating the sample about a vertical axis in the plane of its surface, possible systematic errors resulting from differences in M_8_ and M_11_ are cancelled.

Another advantage of a double reflection from the sample is that the beam is not reversed side to side. When a single reflection is used and the detector swung from the straight through, sample-out position to the reflected light position to make an absolute reflectance measurement, the beam is reversed side to side on the detector. Any nonuniformities in the detector or intervening optics will introduce a systematic error into the measurement. In a double reflection the beam is reversed twice so this error is eliminated. A similar argument can be made for the plane of polarization if it is not in the vertical direction.

**Figure 11:**
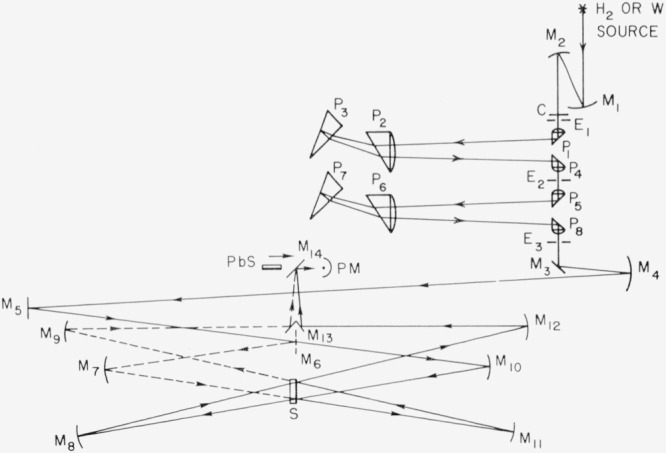
Schematic diagram of absolute reflectometer for use in the ultraviolet and visible wavelength regions employing principle illustrated in figure 9: sample S, mirrors M, prisms P, lead sulfide detector *PbS*, photomultiplier detector PM, slits E, chopper C, hydrogen *H*_2_ or tungsten W sources.

The reflectometer shown in figure 10 uses mirror optics entirely so that measurements can be made from 0.3 to 30 *μ*m. A double-pass monochromator is used to minimize light scattering in the infrared from wavelengths shorter than that used. For ultraviolet measurements double passing is less effective and a second absolute reflectometer shown in figure 11 employs a double monochromator. This reflectometer also has fewer reflections. The angle of incidence for both instruments is about 5°. For unpolarized light the reflectance at 5° differs from that at normal incidence by less than one part in 10^4^ for all real materials. A third absolute reflectometer consisting of a one-meter grating monochromator, a single sample reflection and a rotatable sodium salicylate-coated light pipe-photomultiplier detector system is used for measurements in the vacuum ultraviolet [20].

### D. FECO Scanning Interferometer

**Figure 12:**
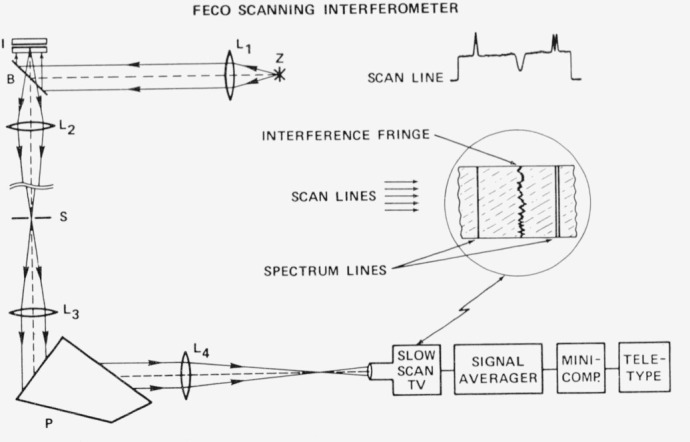
Schematic diagram of FECO Scanning Interferometer. The image detected by the slow scan TV camera is shown at right center and a single scan line is shown above it: interferometer I, xenon arc Z, lenses L, beam splitter B, silt S and prism P.

In order to compare observed values of scattering and absorption caused by surface irregularities with theoretical predictions, a direct measurement of surface structure is required. The FECO Scanning Interferometer which was developed by J. M. Bennett of our laboratory [10, 20] is shown schematically in figure 12. Studies of individual irregularities and statistical surface characterization can be performed with this instrument. The sample to be measured forms the top half of the interferometer I and is illuminated in reflection by xenon arc lamp Z. FECO fringes contouring the surface are formed in the focal plane of a slow scan TV camera along with reference wavelengths for determining fringe position as indicated in the right portion of the figure. The output of the TV camera is fed to a computer system to provide the height distribution function, slope distribution function and autocovariance function characterizing the surface irregularities. The height of the spectrometer slit defines the 1 mm length of the long narrow section of the sample which is evaluated; the lateral resolution of the system is 2 *μ*m. By translating the sample sideways the projected slit can be made to sweep out an area on the sample surface for statistical characterization.

## IV. Results

### A. Scalar Scattering

**Figure 13:**
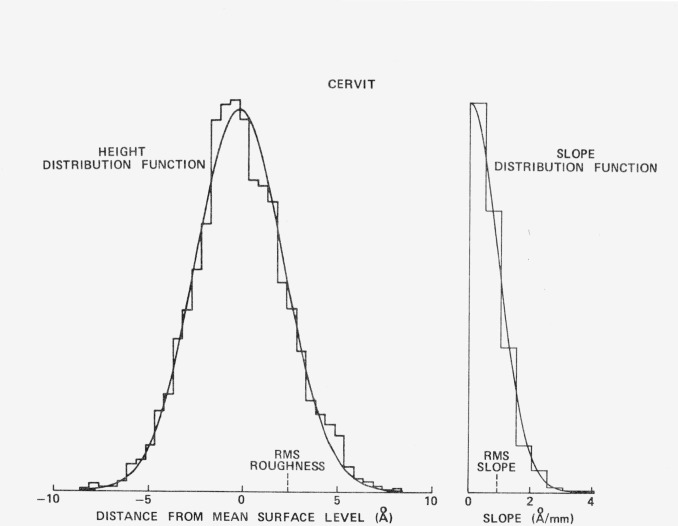
Height and slope distribution functions for a superpolished Cervit optical flat obtained with the FECO Scanning Interferometer. The smooth curves are Gaussians that have the same area under the curves as the measured histograms.

**Figure 14:**
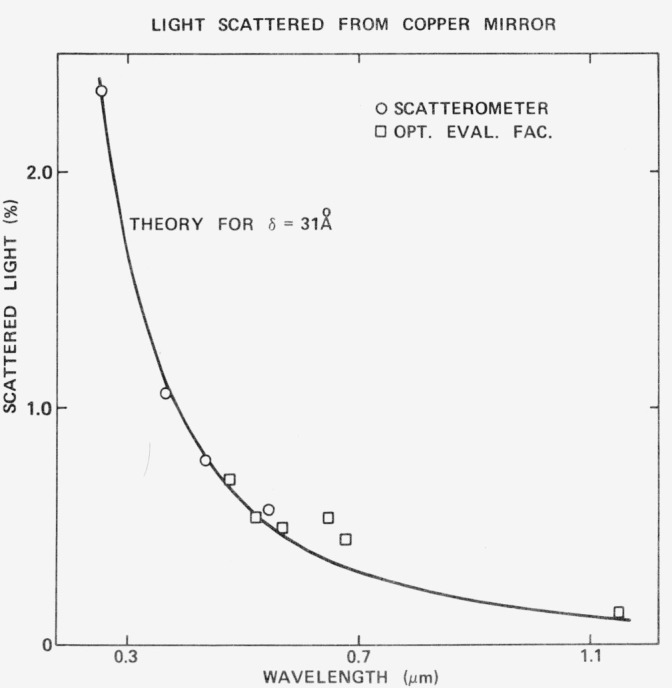
Scattered light as a function of wavelength for a polished copper mirror. The circles represent the hemispherical scatter measurements taken on the Scatterometer and the squares measurements taken on the Optical Evaluation Facility. The solid curve is the hemispherical scattering predicted by the scalar scattering theory.

The height and slope distributions for a superpolished Cervit flat [10], as determined using the FECO Scanning Interferometer are shown in figure 13. A Gaussian curve having the same area as the histogram giving the experimental data is also shown. The fit is quite good so that eq (3) can be used to calculate the scattering level. Both glass and polished metal surfaces examined thus far do have height distribution functions that are nearly Gaussian [10], although their autocovariance functions are strongly non-Gaussian [10]. Figure 14 shows the light scattered from a polished copper mirror. The circles represent data taken with the NWC Scatterometer and the squares data that are taken with the Optical Evaluation Facility. The agreement between the two instruments is quite good. The solid line is the scattering level predicted by eq (3) for a 31 Å rms surface. Agreement here is also quite good.

The rms roughness of an optically flat surface can be determined independently by using the FECO Scanning Interferometer. A comparison of values for polished glass surfaces [22] obtained from scattered light measurements and FECO interferometry is given in table I. Agreement again is quite good.

For some polished metal samples the agreement between FECO and scattered light measurements is not as good as that shown in table I. Reasons may be that the surface is gently rolling with a long autocovariance length, that optical excitation and reemission of surface plasmons is occurring, that the height distribution function is not quite Gaussian, that contributions to scattered light are being made by irregularities too small to be resolved by the FECO system, that a significant contribution to the scattering process is being made by scratches, etc., or for some other as yet unsuspected reason. For most optical surfaces, however, the agreement between predicted and actual scattering behavior in the visible and ultraviolet regions based on eq. (3) is excellent.

**Figure 15:**
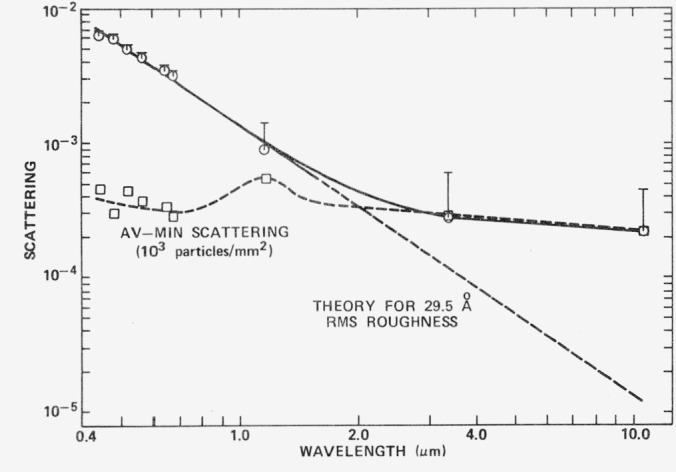
Scattering from an aluminized polished dense flint glass mirror. The open circles are minimum scattering values obtained for 10 points across the surface; averages of the 10 values are indicated by the crossbars. The open squares are the differences between these average and minimum values. The solid line is a fit to the minimum values and the straight dashed line gives scattering levels predicted by eq (4).

At infrared wavelengths the agreement between experimentally observed scattering levels and those predicted by eq (3) may be excellent or may be like that illustrated in figure 15. The circles represent the minimum scattering levels obtained from measurements at ten positions on an aluminized polished dense flint glass sample. The bars represent average values. The difference between the average and minimum values is indicated by the square points. Since an exponential becomes a straight line on log-log paper, the predictions of eq (3) are represented by the diagonal line. In the visible and ultraviolet regions there is good agreement between experiment and the theoretical predictions for a 29.5 Å rms surface. However, in the infrared the experimental points begin to deviate from theory and approach a nearly constant value which coincides with the variations from point to point on the surface. When this surface was examined under a microscope using oblique illumination, about 10^3^ particles/mm^2^ were observed as scattering centers as well as various sleaks and scratches. We believe that the additional infrared scattering observed on this sample resulted from a change in dominant scattering mechanism from microirregularities to scratches and particulates. Scattering from both particulates and scratches should be nearly constant in the visible region where both would be expected to have dimensions larger than a wavelength. The variation from point to point on the surface of this sample is an order of magnitude less than the scattering caused by microirregularities, which suggests that scattering from scratches and particulates is negligible in the visible and ultraviolet. However, with increasing wavelength microirregularity scattering decreases and the scratch and particulate scattering becomes dominant.

**Figure 16:**
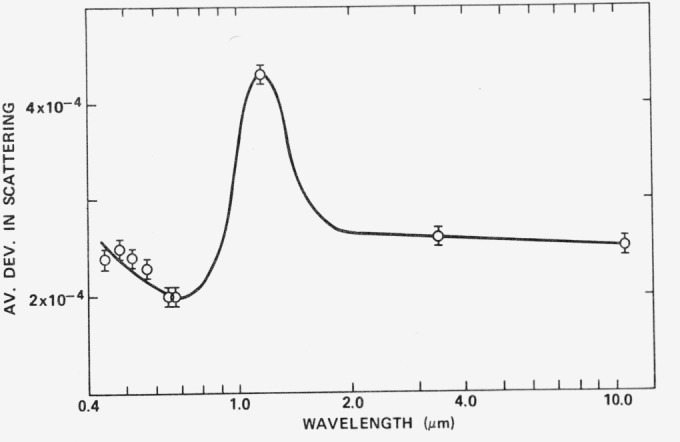
Average deviation in scattering from 10 points on the dense flint sample of figure 15. Values obtained from same measurements as those shown in figure. 15.

The slight hump in the scattering curve shows up even more prominently when the average deviation in scattering from this sample is plotted in figure 16. A resonance would be expected for particulate scattering when the wavelength nearly equals the particle diameter [4] as seen in figure 1. If the particulate has an index of refraction of 1.5 and is not strongly absorbing, this resonance should occur at a particle diameter to wavelength ratio of about 0.7 according to figure 1, or at a wavelength of 1.4 *μ*m if a 1 *μ*m particle diameter is assumed. The observed resonance in figure 16 is in this wavelength range and may thus be caused by dust particles on the mirror surface, which if largely SiO_2_ would have an index of about 1.5. That typical dust particles in our laboratory have a diameter of about 1 *μ*m is shown by assembling the FECO Scanning Interferometer. Dust particles are used as spacers and the minimum order of interference which normally is possible is about 4, which would correspond to a 1 *μ*m diameter dust particle. From these data we conclude that scattering from dust and scratches may be important in the infrared, but for good clean optical surfaces microirregularity scattering is the dominant scattering mechanism in the visible and ultraviolet wavelength regions.

### B. Surface Plasmon Excitation

**Figure 17:**
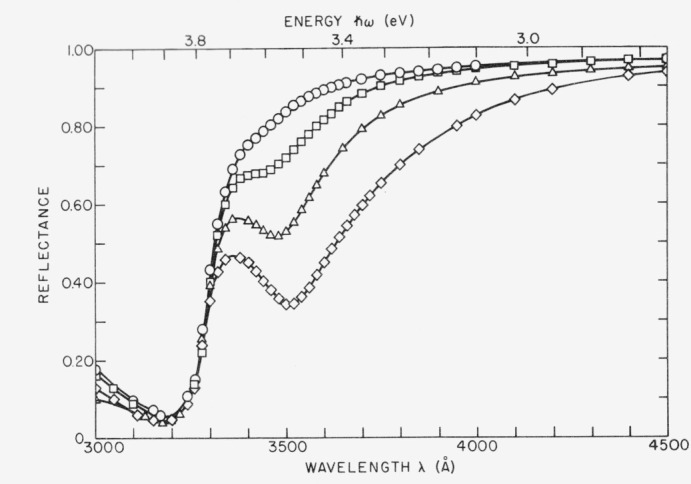
Normal incidence reflectance of silver for surfaces of various roughnesses. As the roughness increases the reflectance decreases.

**Figure 18:**
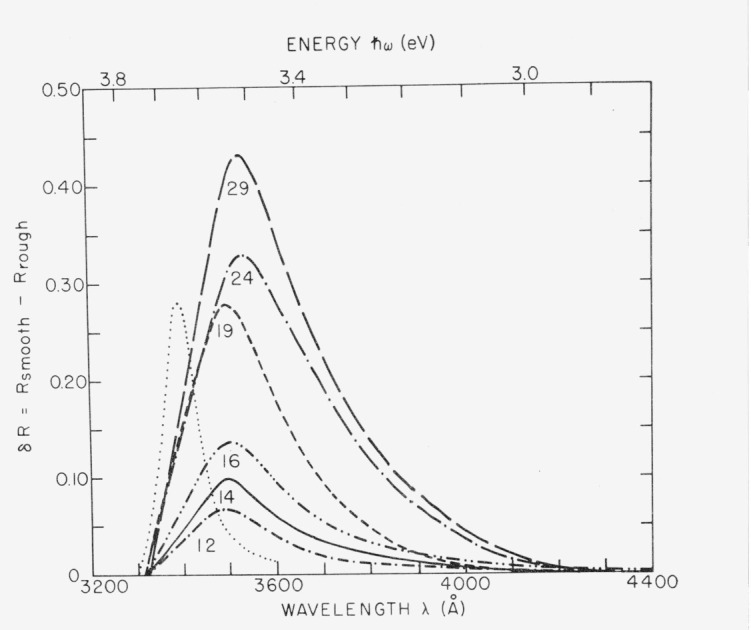
Optical absorption by rough silver coated surfaces resulting from surface plasmon excitation. The roughness value in Å for each surface is given near the absorption peak. The dotted line shows the expected position of the absorption peak if there were no retardation effects.

Microirregularities can give rise to scattered light not only through classical diffraction but also through surface plasmon excitation and reemission. This mechanism also introduces additional absorption. Figure 17 shows the near ultraviolet reflectance of silver deposited on substrates of varying roughness. A large increase in absorption with increasing roughness is observed at wavelengths slightly longer than the limiting wavelength for surface plasmon excitation, which for silver occurs at about 3390 Å where *ϵ*_1_ = −1. By subtracting the rough surface reflectance from the smooth surface reflectance the additional absorption introduced by the rough surface is obtained. The results for various surface roughnesses are shown in figure 18. The absorption peak occurs between 3500 and 3550 Å for this type of rough surface, which was produced by evaporating increasingly thick films of CaF_2_ on an initially smooth substrate. Roughness values are shown under each absorption curve; the initial roughness of the fused quartz substrate was 12 Å rms. A comparison of roughness values for CaF_2_-coated substrates as determined from light scattering measurements and interferometrically is given in table II. The slightly lower values obtained from FECO measurements for the thicker CaF_2_ films probably result from contributions to scattered light by crystallites too small to be resolved by the interferometer. The agreement between these two independent measurement techniques is still quite good.

The shape of the absorption curve which would be predicted if plasma excitation occurred at *ϵ*_1_ = −1 is given by the dotted curve in figure 18. A silver sulfide film only 9 Å thick would be enough to cause the observed shift in the resonance to longer wavelengths, but careful ellipsometric studies of the growth of silver sulfide on silver [23, 24] have established that the observed shift does not result from silver sulfide contamination. It probably occurs because the momentum required from the surface to excite a plasmon at the limiting wavelength would be much larger than that for plasmons in the retardation region (see fig. 5).

**Figure 19:**
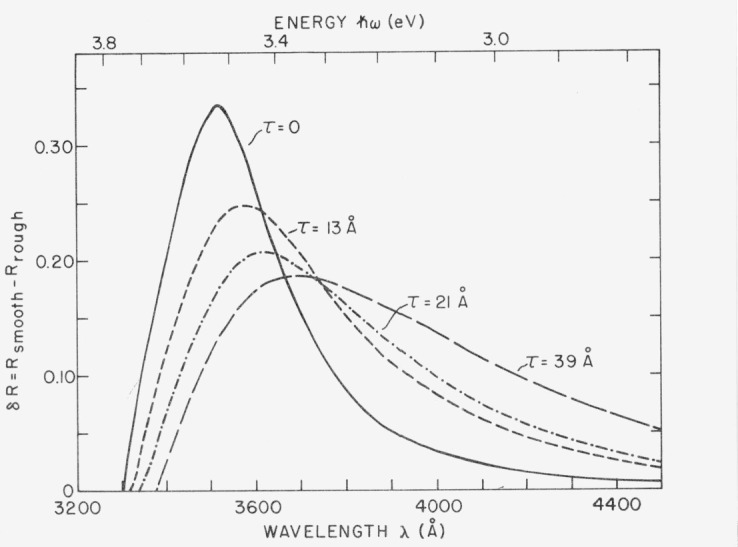
Optical absorption by tarnished silver surfaces resulting from surface plasmon excitation. The τ values give the thicknesses of the different silver sulfide tarnish films.

The growth of a surface film such as Ag_2_S on the silver surface will move the plasma resonance to longer wavelengths and, if the film is absorbing, will damp it. Figure 19 shows the measured change in plasmon-induced absorption of silver on a rough surface as the sulfide film grows [23]. Sulfide film thicknesses *τ* were determined ellipsometrically. They could also be computed from the shift in peak position of the absorption resulting from changes in the dispersion relation for a metal-thin film-air interface. The average difference between the values of *τ* obtained by these two techniques was only 1 Å in this case.

**Figure 20:**
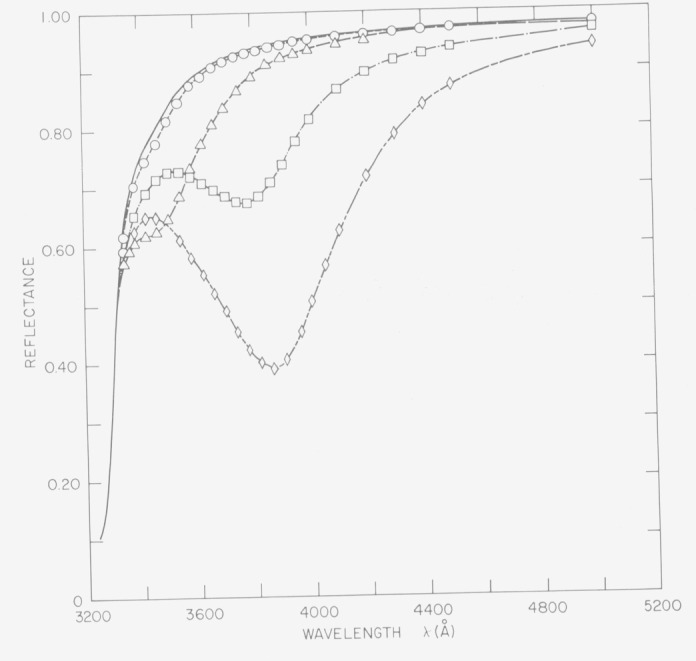
Normal incidence reflectance of slightly rough (circles) and moderately rough (triangles) silver surfaces. The reflectances of the same surfaces after being coated with a 250 Å thick layer of Al2O3 are shown by the squares and diamonds, respectively.

If the surface film is nonabsorbing, it not only moves the absorption peak to longer wavelengths but may also significantly enhance the absorption by surface plasmons [25]. In figure 20 the solid line represents the reflectance of silver deposited on a very smooth substrate and the circles and triangles silver deposited on a slightly rough and moderately rough substrate. When a 250 Å thick film of Al_2_O_3_ was deposited on the silver the slightly rough surface had the reflectance indicated by the squares and the moderately rough surface that indicated by the diamonds. Not only do the reflectance minima move to longer wavelengths but they are also greatly enhanced. This effect is of considerable importance for aluminized mirrors used in the vacuum ultraviolet. The protective MgF_2_ overcoat used to prevent oxidation of the aluminum coating can significantly reduce mirror reflectance above 1600 Å through enhanced absorption by surface plasmons unless the mirror surface is extremely smooth. A similar effect is seen for silvered quartz back surface reflectors. The mirror often has a golden appearance because enhanced surface plasmon absorption reduces the reflectance in the blue region of the spectrum. By intentionally roughening the back surface the reflectance of silver in the blue region of the spectrum can be reduced nearly to zero.

**Figure 21:**
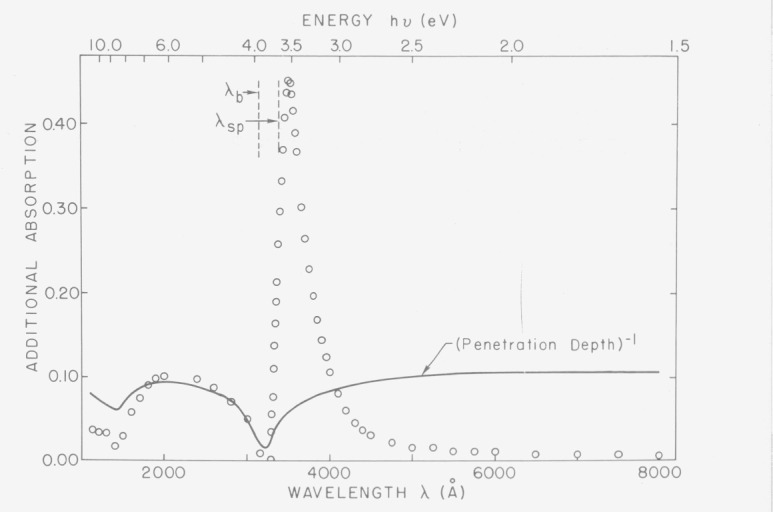
Comparison of the roughness induced increase in absorption at normal incidence for silver and the reciprocal of the optical penetration depth. Note the agreement in minima both at 3200 Å and 1400 Å.

Although most of the increase in absorption observed on rough silver surfaces is found at wavelengths longer than the limiting wavelength for surface plasmon excitation, some increase in absorption is also observed at shorter wavelengths. Figure 21 shows the increase in absorption extending from the visible region into the vacuum ultraviolet. Also plotted is a line proportional to the reciprocal of the optical penetration depth. Although this line has no apparent relation to the observed absorption to the right of the surface plasmon limiting wavelength *λ_sp_* it does fit that to the left of this wavelength rather well. In particular it reproduces both minima in the ultraviolet reflectance data at about 1400 Å and 3200 Å. In addition, it has approximately the exponential dependence to the left of the volume plasmon wavelength *λ_b_* found experimentally by Hunderi and Beaglehole [26] and verified by these measurements. The magnitude of this additional absorption for *λ*<*λ_b_* for samples with varying degrees of roughness is directly proportional to the ration between the rms roughness and the penetration depth. The intensity penetration depth for Ag in the interband region is about 150 Å. A 20 Å rms surface roughness will have a peak to valley height of irregularities of 50 Å or more, roughly a third of the penetration depth. Rougher surfaces will have irregularities which are an even larger fraction of the penetration depth. The band structure of a material derives its character from the regular crystalline array of the atoms. Modification of this array near the surface of the crystal should modify the band structure of the material somewhat. A possible explanation for this additional absorption is that the surface roughness introduces a perturbation of the band structure at the surface of the crystal where its optical behavior is determined. Interband transitions would be expected to be significantly affected by this perturbation, hence the anomalous absorption in the interband region for silver. Intraband transitions should be much less affected, as appears to be true for silver in the free carrier region.

**Figure 22:**
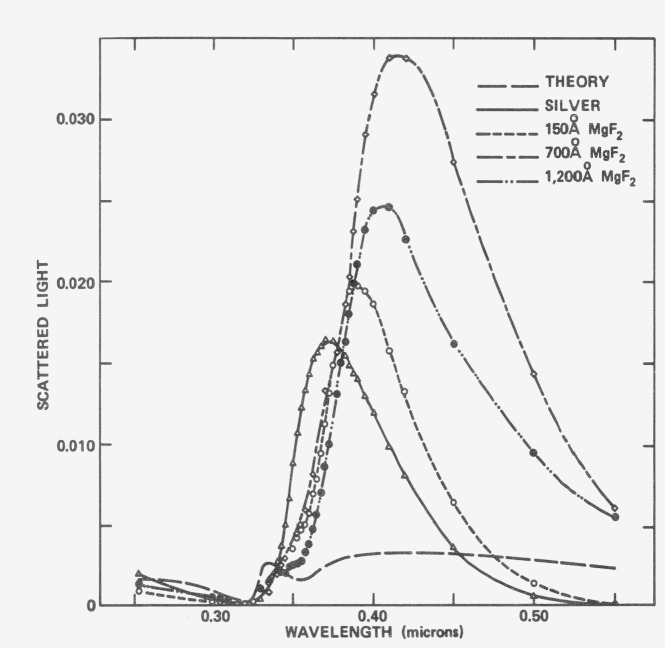
The long-dashed curve is the wavelength dependence of the ratio of the total scattered light to the incident light for a “rough” (21.5 *Å* rms) silver surface calculated from scalar scattering theory. Other curves are the difference between measured values and the calculated curve; solid curve is for a bare silver surface, and other dashed curves are for silver surfaces coated with the indicated thicknesses of magnesium fluoride.

In addition to additional absorption, surface plasmon excitations can also cause significant increases in scattered light [18]. Figure 22 shows the effect for silver deposited on a CaF_2_-roughened substrate having *δ* = 21.5 A. The scattering level calculated for this surface from equation (3) is shown by the long-dashed line. The triangles and solid line show the additional scattering which was observed on the uncoated silver surface. When the surface was coated with various thicknesses of MgF_2_ the scattering level rose again and then declined as a result of an interference effect. The peak scattering level was over an order of magnitude higher than that predicted from classical scattering theory for this relatively smooth optical surface.

### C. Mechanical Abrasion and Surface Damage

**Figure 23:**
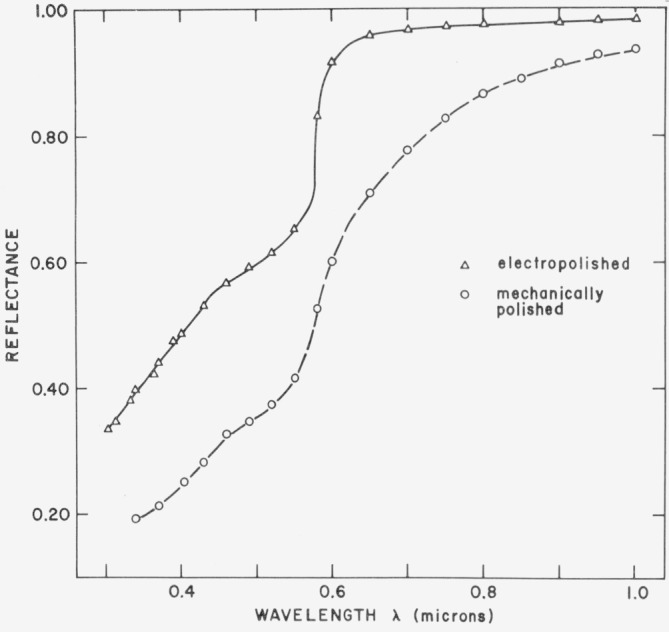
Reflectance of electropolished copper (triangles) and mechanically polished copper (circles) from 0.3 to 1.0 μm. Both samples were cut from the same high-purity ingot.

Figure 23 shows the decrease in reflectance of bulk copper caused by mechanical abrasion [27]. Both copper samples were cut from the same OFHC copper block; one was electropolished, and the other mechanically polished on a silk-covered pitch lap. Both surfaces were optically flat and the electropolished data, which is in good agreement with other optical measurements on high quality bulk copper surfaces and copper films evaporated under good deposition conditions, is representative of the intrinsic reflectance spectrum of copper. The mechanically polished surface has a reflectance spectrum which differs in shape from that of the electropolished sample and is lower by as much as a factor of two. Differences in reflectance persist to 30 *μ*m in wavelength, the limit of the measurements, but are largest in the visible and ultraviolet regions.

**Figure 24:**
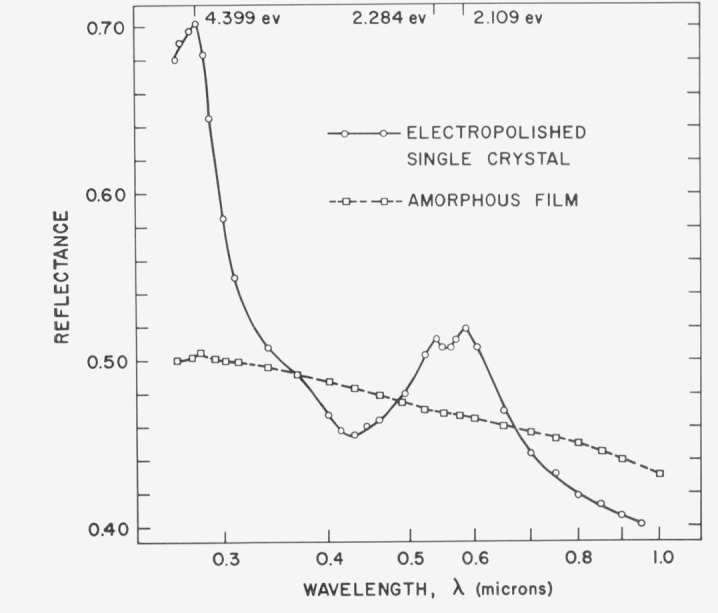
Reflectance of electropolished germanium (circles) and an amorphous germanium film (squares) from 0.26 to 1.0 μm.

Germanium is an even more graphic example of the importance of structure on the optical properties of absorbing materials. In the visible and ultraviolet region it is highly absorbing, strong interband transitions are occurring and the casual observer would think it was a metal. Germanium can be deposited in thin films either in a crystalline or an amorphous state. The reflectance spectra for both cases [28] are shown in figure 24. It is not obvious from the optical spectra that we are even dealing with the same material. However, the amorphous film can be recrystallized by annealing at high temperatures and its reflectance curve then reverts to that for the crystalline case.

The crystalline structure of evaporated metal films is quite complex and its effect on the optical properties is a story in itself. Sputtered metal films are even more complicated and it has been believed that the sputtering process is so uncontrollable that the highest reflectance metal films could not be obtained using it. Sputtered silver, aluminum, and copper films have now been produced [29, 30] which have essentially the same reflectance as those produced by evaporation.

**Figure 25:**
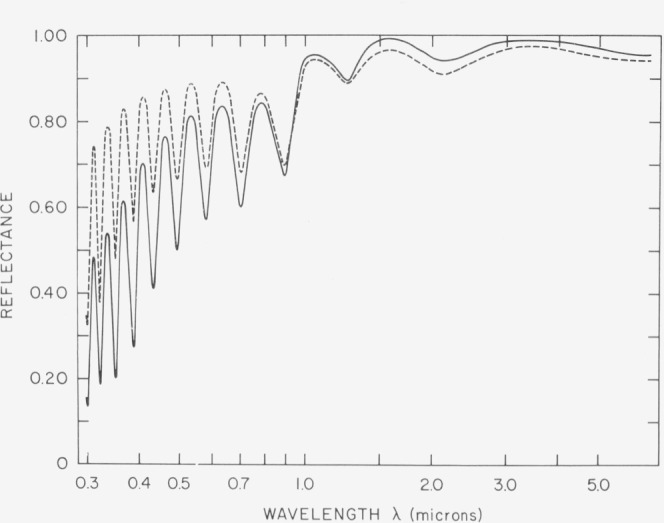
Measured reflectance (solid curve) and calculated reflectance (dashed curve) for an 8869 *Å* film of lead fluoride on aluminum. The optical constants used in the calculations were determined from measurements on evaporated films of the two materials.

The kind of difficulty which is typically found in experimentally obtaining the theoretical optical properties of overcoated metal films is illustrated in figure 25. In this case an aluminum film was deposited by evaporation and then overcoated with a lead fluoride film [31]. The dashed line shows the reflectance spectrum which should have been obtained based on the optical constants of the two materials. The solid line shows that actually obtained. The difference is probably due in large part to the optical excitation of surface plasmons. In aluminum the *ϵ*_1_ curve has a much more gradual shape than for silver, and surface plasmon effects are thus important over a much larger wavelength range. Other factors (which probably were not important) were conventional scattering caused by particulates, and surface roughness and structure-related conventional absorption in both the metal and the dielectric. Notice that the discrepancy between predicted and actual optical performance becomes larger as we go to shorter wavelengths. This example is typical of the problems faced by the optical designer in the visible and ultraviolet regions of the spectrum.

## V. Conclusions

Surface irregularities and crystalline order strongly influence both light scattering and absorption of metal surfaces. These effects are more pronounced in the ultraviolet than at longer wavelengths. Although scattering from isolated irregularities such as dust, scratches and other surface blemishes occurs, it is typically much less important for optically polished surfaces in the visible and ultraviolet regions than scattering from closely spaced microirregularities. In addition to classical scattering from these microirregularities, which if polarization and angular dependence are ignored can be calculated from scalar scattering theory, scattering resulting from surface plasmon excitation and reemission can be very important. It is enhanced by a nonabsorbing dielectric coating, and in limited wavelength regions can exceed classical scattering levels by over an order of magnitude. Surface plasmon excitation also causes additional absorption, which is also enhanced by a dielectric coating. This effect is particularly important in the vacuum ultraviolet where aluminum, for example, must be coated with a nonabsorbing dielectric such as magnesium fluoride to prevent oxidation. Crystalline disorder at the metal surface introduces significant additional absorption, presumably both through surface plasmon excitation and band structure modification. For optimum optical performance of metal surfaces in the visible and ultraviolet regions of the spectrum it is thus essential that they have an absolute minimum of surface roughness and as undistorted a lattice structure as possible.

## Figures and Tables

**Table I:** Comparison of rms roughness values of polished glass surfaces obtained from FECO interferometry and from scattered light measurements

Sample	*δ*_FECO_ (Å)	*δ*_SCAT_ (Å)
ULE Quartz	10.2	8.4
ULE Quartz	12.6	9.9
ULE Quartz	12.7	12.5
Fused Quartz	11.0	10.2
Fused Quartz	16.7	16.4
Fused Quartz	17.3	17.3
BK-7 Glass	19.3	19.1

**Table II:** Comparison of rms roughness values for superpolished optical flats coated with evaporated calcium fluoride films of various thicknesses as determined from FECO interferometry and scattered light measurements

CaF_2_ Thickness (Å)	*δ*_FECO_ (Å)	*δ*_SCAT_ (Å)
0	11	10
875	12	13
1750	16	19
2625	19	26

## Footnotes

1 Figures in brackets indicate the literature references at the end of this paper.
